# A Phase I/II Clinical Trial to evaluate the efficacy of baricitinib to prevent respiratory insufficiency progression in onco-hematological patients affected with COVID19: A structured summary of a study protocol for a randomised controlled trial

**DOI:** 10.1186/s13063-021-05072-4

**Published:** 2021-02-05

**Authors:** G. Moreno-González, A. Mussetti, A. Albasanz-Puig, I. Salvador, A. Sureda, C. Gudiol, R. Salazar, M. Marin, M. Garcia, V. Navarro, I. de la Haba Vaca, E. Coma, G. Sanz-Linares, X. Dura, S. Fontanals, G. Serrano, C. Cruz, R. Mañez

**Affiliations:** 1 Intensive Care Department, Hospital Universitari de Bellvitge, L’Hospitalet de LL., Barcelona, Spain; 2 Bellvitge Biomedical Research Institute (IDIBELL), L’Hospitalet de LL., Barcelona, Spain; 3 Clinical Hematology Department, Institut Català d’ Oncologia, L’Hospitalet de LL., Barcelona, Spain; 4 Infectious Disease Department, Hospital Universitari de Bellvitge, L’Hospitalet de LL., Barcelona, Spain; 5 Spanish Network for Research in Infectious Disease (REIPI), Instituto de Salud Carlos III, Madrid, Spain; 6 Immunology Department, Hospital Universitari de Bellvitge, L’Hospitalet de LL., Barcelona, Spain; 7 Barcelona University, Barcelona, Spain; 8 Clinical Oncology Department, Institut Català d’ Oncologia, L’Hospitalet de LL., Barcelona, Spain; 9 Clinical Trials Unit, Institut Català d’ Oncologia, L’Hospitalet de LL., Barcelona, Spain; 10 Oncology Emergency Unit, Institut Català d’ Oncologia, L’Hospitalet de LL., Barcelona, Spain; 11 Pharmacology Department, Institut Català d’ Oncologia, L’Hospitalet de LL., Barcelona, Spain; 12 Palliative Department, Institut Català d’ Oncologia, L’Hospitalet de LL., Barcelona, Spain

**Keywords:** COVID19, randomised controlled trial, baricitinib, jak inhibitors, respiratory insufficiency, oncological patients

## Abstract

**Objectives:**

Baricitinib is supposed to have a double effect on SARS-CoV2 infection. Firstly, it reduces the inflammatory response through the inhibition of the Januse-Kinase signalling transducer and activator of transcription (JAK-STAT) pathway. Moreover, it reduces the receptor mediated viral endocytosis by AP2-associated protein kinase 1 (AAK1) inhibition. We propose the use of baricinitib to prevent the progression of the respiratory insufficiency in SARS-CoV2 pneumonia in onco-haematological patients. In this phase Ib/II study, the primary objective in the safety cohort is to describe the incidence of severe adverse events associated with baricitinib administration. The primary objective of the randomized phase (baricitinib cohort *versus* standard of care cohort) is to evaluate the number of patients who did not require mechanical oxygen support since start of therapy until day +14 or discharge (whichever it comes first). The secondary objectives of the study (only randomized phase of the study) are represented by the comparison between the two arms of the study in terms of mortality and toxicity at day+30. Moreover, a description of the immunological related changes between the two arms of the study will be reported.

**Trial design:**

**The trial is a phase I/II study** with a safety run-in cohort (phase 1) followed by an open label phase II randomized controlled trial with an experimental arm compared to a standard of care arm.

**Participants:**

The study will be performed at the Institut Català d’Oncologia, a tertiary level oncological referral center in the Catalonia region (Spain). The eligibility criteria are: patients > 18 years affected by oncological diseases; ECOG performance status < 2 (Karnofsky score > 60%); a laboratory confirmed infection with SARS-CoV-2 by means of real -time PCR; radiological signs of low respiratory tract disease; absence of organ dysfunction (a total bilirubin within normal institutional limits, AST/ALT≤2.5 X institutional upper limit of normal, alkaline phosphatase ≤2.5 X institutional upper limit of normal, coagulation within normal institutional limits, creatinine clearance >30 mL/min/1.73 m^2^ for patients with creatinine levels above institutional normal); absence of HIV infection; no active or latent HBV or HCV infection. The exclusion criteria are: patients with oncological diseases who are not candidates to receive any active oncological treatment; hemodynamic instability at time of study enrollment; impossibility to receive oral medication; medical history of recent or active pulmonary embolism or deep venous thrombosis or patients at high-risk of suffering them (surgical intervention, immobilization); multi organ failure, rapid worsening of respiratory function with requirement of fraction of inspired oxygen (FiO_2_) > 50% or high-flow nasal cannula before initiation of study treatment; uncontrolled intercurrent illness (ongoing or severe active infection, symptomatic congestive heart failure, unstable angina pectoris, cardiac arrhythmia, or psychiatric illness/social situations that would limit compliance with study requirements); allergy to one or more of study treatments; pregnant or breastfeeding women; positive pregnancy test in a pre-dose examination. Patients should have the ability to understand, and the willingness to sign, a written informed consent document; the willingness to accept randomization to any assigned treatment arm; and must agree not to enroll in another study of an investigational agent prior to completion of Day +28 of study.

An electronic Case Report Form in the Research Electronic Data Capture (REDCap) platform will be used to collect the data of the trial.

Removal from the study will apply in case of unacceptable adverse event(s), development of an intercurrent illness, condition or procedural complication, which could interfere with the patient’s continued participation and voluntary patient withdrawal from study treatment (all patients are free to withdraw from participation in this study at any time, for any reasons, specified or unspecified, and without prejudice).

**Intervention and comparator:**

Treatment will be administered on an inpatient basis. We will compare the experimental treatment with baricitinib plus the institutional standard of care compared with the standard of care alone. During the phase I, we will define the dose-limiting toxicity of baricitinib and the dose to be used in the phase 2 part of the study. The starting baricitinib dose will be an oral tablet 4 mg-once daily which can be reduced to 2 mg depending on the observed toxicity. The minimum duration of therapy will be 5 days and it can be extended to 7 days. The standard of care will include the following therapies. Antibiotics will be individualized based on clinical suspicion, including the management of febrile neutropenia. Prophylaxis of thromboembolic disease will be administered to all participants. Remdesivir administration will be considered only in patients with severe pneumonia (SatO_2_ <94%) with less than 7 days of onset of symptoms and with supplemental oxygen requirements but not using high-flow nasal cannula, non-invasive or invasive mechanical ventilation or extracorporeal membrane oxygenation (ECMO).

In the randomized phase, tocilizumab or interferon will not be allowed in the experimental arm. Tocilizumab can be used in patients in the standard of care arm at the discretion of the investigator. If it is prescribed it will be used according to the following criteria: patients who, according to his baseline clinical condition, would be an ICU tributary, interstitial pneumonia with severe respiratory failure, patients who are not on mechanical ventilation or ECMO and who are still progressing with corticoid treatment or if they are not candidates for corticosteroids. Mild ARDS (PAFI <300 mmHg) with radiological or blood gases deterioration that meets at least one of the following criteria: CRP >100mg/L D-Dimer >1,000μg/L LDH >400U/L Ferritin >700ng/ml Interleukin 6 ≥40ng/L. The use of tocilizumab is not recommended if there are AST/ALT values greater than 10 times the upper limit of normal, neutrophils <500 cells/mm3, sepsis due to other pathogens other than SARS-CoV-2, presence of comorbidity that can lead to a poor prognosis, complicated diverticulitis or intestinal perforation, ongoing skin infection. The dose will be that recommended by the Spanish Medicine Agency in patients ≥75Kg: 600mg dose whereas in patients <75kg: 400mg dose. Exceptionally, a second infusion can be assessed 12 hours after the first in those patients who experience a worsening of laboratory parameters after a first favourable response. The use of corticosteroids will be recommended in patients who have had symptoms for more than 7 days and who meet all the following criteria: need for oxygen support, non-invasive or invasive mechanical ventilation, acute respiratory failure or rapid deterioration of gas exchange, appearance or worsening of bilateral alveolar-interstitial infiltrates at the radiological level. In case of indication, it is recommended: dexamethasone 6mg/d p.o. or iv for 10 days or methylprednisolone 32mg/d orally or 30mg iv for 10 days or prednisone 40mg day p.o. for 10 days.

**Main outcomes:**

Phase 1 part: to describe the toxicity profile of baricitinib in COVID19 oncological patients during the 5-7 day treatment period and until day +14 or discharge (whichever it comes first).

Phase 2 part: to describe the number of patients in the experimental arm that will not require mechanical oxygen support compared to the standard of care arm until day +14 or discharge (whichever it comes first).

**Randomisation:**

For the phase 2 of the study, the allocation ratio will be 1:1. Randomization process will be carried out electronically through the REDcap platform (https://www.project-redcap.org/)

**Blinding (masking):**

This is an open label study. No blinding will be performed.

**Numbers to be randomised (sample size):**

The first part of the study (safety run-in cohort) will consist in the enrollment of 6 to 12 patients. In this population, we will test the toxicity of the experimental treatment. An incidence of severe adverse events grade 3-4 (graded by Common Terminology Criteria for Adverse Events v.5.0) inferior than 33% will be considered sufficient to follow with the next part of the study. The second part of the study we will perform an interim analysis of efficacy at first 64 assessed patients and a definitive one will analyze 128 assessed patients. *Interim* and definitive tests will be performed considering in both cases an alpha error of 0.05. We consider for the control arm this rate is expected to be 0.60 and for the experimental arm of 0.80. Considering this data, a superiority test to prove a difference of 0.20 with an overall alpha error of 0.10 and a beta error of 0.2 will be performed. Considering a 5% of dropout rate, it is expected that a total of 136 patients, 68 for each study arm, will be required to complete study accrual.

**Trial Status:**

Version 5.0. 14^th^ October 2020

Recruitment started on the 16^th^ of December 2020. Expected end of recruitment is June 2021.

**Trial registration:**

AEMPs: 20-0356

EudraCT: 2020-001789-12, https://www.clinicaltrialsregister.eu/ctr-search/search (Not publically available as Phase I trial)

Clinical trials: BARCOVID19, https://www.clinicaltrials.gov/ (In progress)

**Full protocol:**

The full protocol is attached as an additional file, accessible from the Trials website (Additional file 1). In the interest in expediting dissemination of this material, the familiar formatting has been eliminated; this Letter serves as a summary of the key elements of the full protocol.”

**Supplementary Information:**

The online version contains supplementary material available at 10.1186/s13063-021-05072-4.

## Supplementary Information

**Additional file 1.**

## Data Availability

Not applicable

