# Supplementary material for: A Phase I/II Clinical Trial to evaluate the efficacy of baricitinib to prevent respiratory insufficiency progression in onco-hematological patients affected with COVID19: A structured summary of a study protocol for a randomised controlled trial
Source: Trials. 2021 Feb 5;22:116. doi: 10.1186/s13063-021-05072-4 (PMC7862837; doi:10.1186/s13063-021-05072-4)
Supplement: Supplementary file 1 — Additional file 1. [file 13063_2021_5072_MOESM1_ESM.docx]

**A PHASE I/II CLINICAL TRIAL TO EVALUATE THE EFFICACY OF BARICITINIB TO PREVENT RESPIRATORY INSUFFICIENCY PROGRESSION IN ONCO-HEMATOLOGICAL PATIENTS AFFECTED WITH COVID19**

Protocol Code: BarCOVID19

Version: 5.0 14.oct.2020.

Principal Investigator: Dr. Gabriel Moreno-Gonzalez

EudraCT number: 2020-001789-12

Co-IPs: Dr. Alberto Mussetti, Dr. Iñaki Salvador, Dra. Adaia Albasanz Puig

Study promotor:

Institut Català d’Oncologia

Av Gran Via S/N Km 2,7

L´Hospitalet de Llobregat, Barcelona


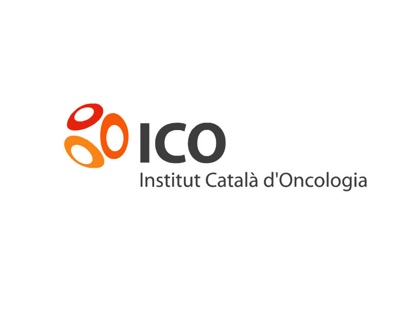

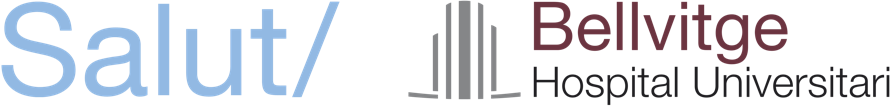


1. **SUMMARY**

Type of request:

Phase I / II clinical trial

Study promoter:

Institut Català d’Oncologia

L´Hospitalet de Llobregat

Barcelona

Trial title:

**A PHASE I/II CLINICAL TRIAL TO EVALUATE THE EFFICACY OF BARICITINIB TO PREVENT RESPIRATORY INSUFFICIENCY PROGRESSION IN ONCO-HEMATOLOGICAL PATIENTS AFFECTED WITH COVID19**

**Protocol code: BarCOVID19**

Principal investigator: Dr. Gabriel J Moreno González

Collaborating investigators: Dr. Alberto Mussetti, Dr. Iñaki Salvador, Dra. Adaia Albasanz-Puig

**Centers participating in the trial:**

Catalan Institute of Oncology of l’Hospitalet

**Ethical Committees that have approved the trial:**

Ethical Committee of Bellvitge University Hospital

**Monitoring:**

UICEC Idibell

**Experimental drug:** Baricitinib

**Phase of the clinical trial:**

1st Stage: Phase I / II.

2nd Stage: Phase II

**Main objective:**

**Part I: Security Cohort:**

To determine the tolerability profile of baricitinib in oncohematological patients with SARS-COV2 infection

**Part II: Phase II trial:**

To determine the efficacy of baricitinib added to the treatment at the discretion of the investigator in the early reduction of the inflammatory response produced by the SARS-COV2 infection and the prevention of the development of severe adult respiratory distress syndrome (ARDS) in oncohematological patients compared to treatment at the discretion of the investigator.

**Secondary objectives:**

1. Compare mortality within the first 30 days from the start of treatment, between the two treatment arms.

2. Compare the toxicity profile between the two treatment arms.

3. Describe the variability of the immune response due to the experimental treatment *versus* the treatment at the investigator's discretion

4. Identify early biomarkers of poor outcome or response to treatment

**Design:**

The study has an open, randomized, two-part design:

Part I: Security Cohort:

Between 6 and 12 patients will be treated sequentially in a single center, to evaluate the toxicity of the experimental regimen.

Part II: Phase II Randomized:

136 patients (68 per arm) will be recruited, in a single center, and will be randomized to receive baricitinib plus the standard treatment at the investigator's discretion or only the standard treatment. An *interim* analysis of efficacy and futility is planned after the recruitment of the first 68 evaluable patients.

**Disease under study:**

Acute respiratory failure due to SARS COV2 virus.

● **Primary endpoint:**

The primary endpoint is the percentage of cancer patients with acute respiratory failure due to COVID19 who have not required mechanical oxygen support from the start of therapy until day +14 or discharge from hospital (whichever occurs first). In particular, percentage of patients with a scale 4 or more of EOSC (see appendix)

**● Study population:**

Patients with a confirmed diagnosis of acute respiratory failure due to SARS COV2 virus and oncohematological tumors

**● Duration of experimental treatment:** 5 - 7 days.

**Schedule and expected completion date:**

Start of study September 01, 2020, end March 31, 2021

**2. INDEX**

1. SUMMARY P2

2. INDEX P4

3. JUSTIFICATION AND OBJECTIVES P6

3.1. Justification P6

4. TEST DESIGN P7

4.1 Part I: Security Cohort: P7

4.2 Part II: Phase II trial: P7

5. OBJECTIVES P8

5.1 Primary objective: P8

5.2 Secondary objectives: P8

6. STUDY TYPE AND DESIGN P8

7. DESCRIPTION OF THE RANDOMIZATION AND STRATIFICATION P8

8. PATIENT SELECTION P8

8.1. Inclusion criteria P8

8.2. Exclusion criteria P9

8.3. Number of expected patients P10

8.4. Expected analysis of withdrawals and dropouts P10

8.4.1. Withdrawal from study treatment P10

8.4.2. Trial withdrawal criteria P10

8.5. Duration of recruitment period P10

9. TREATMENT PLAN P10

9.1. Baricitinib (experimental medication, OLUMIANT ©) P10

9.1.1. Posology P11

9.1.2. Adverse effects: P12

9.1.3. Interactions: P12

9.1.3.1. Dose limiting toxicity P12

9.1.4.1 Unacceptable toxicity P12

9.2. Concomitant treatment P12

9.3. Other medication P13

10. DEVELOPMENT OF THE TRIAL AND EVALUATION OF THE RESPONSE P15

10.1. Study Outline P15

10.1.1. Evaluations before treatment: P15

10.1.2. Assessments during the study (day 2, day 7, day 14 or discharge, day 30): P16

10.2. Obtaining biological samples P16

10.2.1. Samples for future studies P16

10.3. TOXICITY ASSESSMENT P16

10.4. Pharmacokinetic and pharmacodynamic analyzes P16

10.5. Withdrawals and dropouts P16

11. ADVERSE EVENTS P17

11.1. Definitions P17

11.2. Reference safety information P18

11.3. Assessment of adverse events for the safety cohort P18

11.4. Assessment of adverse events for the futility cohort / phase II trial P19

11.5. Follow-up of patients with adverse events P20

11.6. Notification of adverse events P20

11.7. Serious and unexpected adverse reactions P20

11.8. Expedited notification of other relevant safety information P21

11.9. Pregnancy P21

11.10. Report to Researchers P21

12. ETHICAL ASPECTS P21

12.1. General considerations P21

12.1.1 Approval of the protocol by the Ethics Committee for Drug Research. P22

12.2. Informed consent P22

12.3. Confidentiality P23

12.4. Insurance policy P23

13. PRACTICAL CONSIDERATIONS P23

13.1. Investigator responsibilities P23

13.2. Responsibility for the product P24

13.3. File Maintenance P24

13.4. Data collection notebooks P24

13.5. Protocol adherence P24

13.6. Amendments to protocol P24

13.7. Study monitoring P25

13.8. Valuation variables P25

13.8.1. Main variable: P25

13.8.1.1. Part I: Safety cohort (run-in cohort) P25

13.8.1.2. Part II: Randomized Trial P25

13.8.2. Secondary objectives: P25

13.9. Secondary variables P25

14. STATISTICAL METHODS P26

14.1. Randomized Phase II Trial: Sample Size and Main Analysis P26

14.2 Statistical analysis P27

14.2.1. Sample description P27

14.2.2. Defining of analysis populations P27

14.2.3. Phase II main analysis P27

14.2.4 Secondary analyzes P28

**3. JUSTIFICATION**

The new coronavirus SARS-COV-2 is currently challenging the health care systems of our society. This new pandemic represents both a medical and public health care challenge. In fact, while more than 80% of infected people are expected to be asymptomatic, the rest of the patients will present with respiratory symptoms. Of these, 10% will require hospitalization and 1-2% will eventually die (PMID 32109013, PMID 32171076). The number of patients requiring intensive care unit hospitalization is rapidly progressing and logistic problems related to the limited numbers of Intensive Care Unit (ICU) beds or ventilators could severely limit the availability of a sufficient supportive care.

The syndrome generated from SARS-COV-2, called COVID19, is composed of 3 phases. The first phase is the prodromal phase. During 5 to 7 days, patients start to develop mild myalgias, low-grade fever and non-specific symptoms. This phase is followed by the symptomatic phase characterized by high-grade fever and respiratory symptoms (usually dry cough). During this phase, it is supposed that the innate immune system starts to reply to the infection. It generally lasts 6-7 days. After this time interval, the adaptive immune system starts reacting against the virus. At this point, 2 scenarios are possible: 1) an effective, controlled immune reaction able to neutralize the infection; 2) an uncontrolled and excessive immune reaction with development of acute respiratory distress syndrome. In the latter case, it is likely that the patient will develop dyspnea, hypoxia and a possible need of endotracheal intubation. This phase could last between 2-3 weeks before resolution or worsen to multiorgan failure and death (PMID 32103284, PMID 32171076).

Most of the treatments available today are related to phase 1 and 2 of the syndrome (PMID 32152082). In these phases, the aim of the therapy is reducing viral replication. Hopefully, in the future a vaccine will be able to address the disease at population level (PMID 32186952). Our study is focused on preventing the phase 3 of the COVID19. Reducing an excessive inflammatory response is thought to improve a patient's clinical status and avoid the development of ARDS. If this proves to be successful, a significant number of patients will not require an intensive care support reducing the chance of death. Moreover, reducing the number of patients who require an ICU unit will possibly reduce the logistical burden of the disease. This could help in the nationwide management of the syndrome.

Baricitinib is a JaK1/Jak2 inhibitor currently approved for the management of rheumatoid arthritis. It is supposed to have a double biological effect against the virus. First, it could reduce the receptor-mediated viral endocytosis by means of AAK1 inhibition. Secondly, it could reduce the inflammatory cytokines release storm by inhibition of the Jak1/Jak2 pathway. It is known that some patients with severe infection develop a cytokine release syndrome (PMID 32192578). In those patients, the use of tocilizumab has demonstrated that Il-6 signaling inhibition is an strategy to control the disease ChiCTR2000029765. This monoclonal antibody blocks the surface IL-6 receptor avoiding their binding, inhibiting the Il-6 mediated inflammatory response. Baricitinib is a specific Jak1/Jak2 inhibitor leading to inhibition of the IL-6 and IL-1 mediated signaling, but not of those mediated by other JAK quinases. Also, the JAK1 and JAK2 kinases regulate other pro inflammatory molecules like INFα or INFγ and some anti-inflammatory citoquines like IL-10 (PMID 31375130). Some *in vitro* studies have demonstrated that baricitinib regulates the innate inflammatory response reducing the dendritic production of interferons type 1, the B lymphocyte production of IL-6 and reducing the expression of co-stimulatory molecules in dendritic cells derived from macrophages (PMID [30002661](https://www.ncbi.nlm.nih.gov/pubmed/30002661)). Due to the relevance of the interferons in the antiviral response, we believe that baricitinib could be better to tocilizumab in the control of CRS during the SARS-CoV2 infection. Finally, baricitinib blocks the viral endocytosis through the direct effect on AAK1 and GAK kinases (PMID 32032529).

Baricitinib has a half-life of 12hr (PMID 29134648) then a short course of treatment could have few adverse events. Moreover, once stopped the inflammatory response should be re initiated due to the reversible inhibition of Jaks quinases. Finally, due to the fact that baricitinib is a small molecule the mass production and distribution should be shorter and the cost lower compared to other Il-6 inhibitors.

In this phase 1/2 study, we are planning to include oncological patients affected by COVID19 validated by a probe and characterized by radiological evidence of low airways involvement. The experimental arm of the study will receive baricitinib at a dose of 4mg/24 hours for 7 days.

In order to minimize the risk for patients, given that there are no previous toxicity data from baricitinib treatment in onco-hematological patients with COVID19, the study will be divided into two parts.

**4. STUDY DESIGN**
4.1 Part I: Security Cohort: Between 6 and 12 patients will be treated sequentially in a single center, to evaluate the tolerability of the experimental regimen, based on an algorithm in which two possible doses will be evaluated.

4.2 Part II: Phase II trial:

If the safety part I is passed, the randomized phase II trial part will start. This phase will consist in an *interim* analysis of efficacy and futility once the first half of recruitment is completed(see statistics section)

Furthermore, there is currently no biomarker capable of defining which patients will evolve in a unfavorable way or in response to treatment. Conventional inflammation markers such as CRP, ferritin or D-dimer, it has been proposed that IL-6 levels> 50pg / mL could be predictors of poor evolution. These levels are similar to those found in severe influenza infection (PMID 22174866, PMID 19785746) and much lower than those found in septic shock or cytokine release syndrome (PMID 30400775, PMID 21991134, PMID 27049586). In this sense, new biomarkers will be sought in a panel of molecules related to the immune response, inflammation, cell death (necrosis, necroptosis) and fibrosis.

**5.0 OBJECTIVES**

5.1 **Primary Objectives**

- Safety cohort (first 6-12 patients): describe the incidence of severe adverse events during baricitinib administration
- Randomized phase: evaluate the number of patients who did not require mechanical oxygen support (high-flow nasal cannula, CiPAP, endotracheal intubation, ECMO) since start of therapy until day +14 or discharge (whichever it comes first)

5.2 **Secondary Objectives**

- Day +30 mortality comparison between the two arms of the study
- Toxicity comparison between the two arms of the study
- Describe the immunological related changes between the two arms of the study
- Identify biomarkers for early recognition of worst response of treatment failure

**6.0. STUDY TYPE AND DESIGN**

In the first part we will perform a safety run-in cohort with the experimental drug at 4mg or 2mg according to the study design. If a safety dose is identified, we will perform an open label phase II randomized controlled trial with an experimental arm compared to a control arm. We will perform an interim analysis at 50% of recruitment.

**7.0 DESCRIPTION OF THE RANDOMIZATION AND STRATIFICATION**

We will perform a block randomization without stratifications.

**8.0 PATIENT SELECTION**

All patients should meet all the inclusion criteria and none of the exclusion criteria

8.1 **Inclusion Criteria**

- Patients affected by oncological diseases
- Age > 18 years
- ECOG performance status < 2 (Karnofsky > 60%)
- Laboratory (RT-PCR) confirmed infection with SARS-COV2Radiological signs of low respiratory tract disease
- patients on active antitumor treatment who are candidates to endotracheal intubation (NIT1)
- patients defined as oncological patients who are candidates or receiving active chemotherapy without indication to endotracheal intubation due to oncological disease or clinical status (NIT2)
- patients defined as oncological patients who are candidates or receiving active palliative chemotherapy without indication to endotracheal intubation due to oncological disease or clinical status. (NIT3)
- Patients must have normal organ function as defined below:
- total bilirubin within normal institutional limits
- AST(SGOT)/ALT(SGPT)≤2.5 X institutional upper limit of normal
- Alkaline phosphatase ≤2.5 X institutional upper limit of normal
- coagulation within normal institutional limits
- creatinine clearance >30 mL/min/1.73 m^2^ for patients with creatinine levels above institutional normal
- HIV seronegativity; no active or latent HBV or HCV infection
  Ability to understand and the willingness to sign a written informed consent document.
- Willingness of study participant to accept randomization to any assigned treatment arm.
- Must agree not to enroll in another study of an investigational agent prior to completion of Day 28 of study.

8.2 **Exclusion Criteria**

- Patients with oncological diseases who are not eligible to receive any active oncological treatment.
- Hemodynamic instability at time of study screening
- Impossibility to receive oral medication
- Medical history of pulmonary embolism or deep venous thrombosis or patients with high risk of suffering them (surgical intervention, immobilization)
- Multi organ failure
- Rapid worsening of respiratory function with requirement of FiO2 > 50% or high-flow nasal cannula before initiation of study treatment
- Uncontrolled intercurrent illness including, but not limited to, ongoing or severe active infection, symptomatic congestive heart failure, unstable angina pectoris, cardiac arrhythmia, or psychiatric illness/social situations that would limit compliance with study requirements.
- Allergy to one or more of study treatments
- Pregnant or breastfeeding, or positive pregnancy test in a pre-dose examination
- Patients may not be receiving any other investigational agents

**8.3** **Number of patients**
It is estimated that for the first stage, between 6 and 12 patients are necessary. For the second stage (Phase II), 68 more patients will be needed for the intermediate analysis and if it continues, a total of 136 patients will be required

**8.4.1. Withdrawal of study treatment**

A patient will drop out of the study for any of the following reasons:

- If it is considered of interest for the patient to suspend treatment, according to the doctor's criteria.

- If requested by the patient.

- If there is non-compliance with the protocol.

- If it presents an unacceptable toxicity or threat to life.

- Intercurrent illnesses or other reasons that, in the opinion of the investigator, significantly affect the assessment of the clinical status and that require the interruption of treatment

- Rapid respiratory deterioration with high O2 requirements (FIO2> 50%) or NIMV in the first 48h after starting treatment with Baricitinib

If the patient is withdrawn from the study:

1. The reason (s) for abandonment should be documented in the patient's medical record and on the CRD.

2. Every effort will be made to maintain the research program and continue follow-up, even if patients prematurely discontinue protocol treatment and / or no longer visit the participating institution.

**8.4.2. Trial withdrawal criterion**

The criteria for withdrawal from the trial are as follows:

• The patient reaches the end of follow-up (day 30 from inclusion)

• The patient withdraws consent

• Patient death

**8.5. Duration of the recruitment period**

Study recruitment will start from the first day of baricitinib administration. The estimated recruitment period will be between 2 and 3 months (considering the recruitment of 5 patients per week as calculated from the last week of clinical registrations at our center).

**9.0 TREATMENT PLAN**

Investigational Drugs (MI) will be considered in this study: baricitinib

**9.1. Baricitinib (experimental medication, OLUMIANT ©)**

**9.1.1. Posology**

**Tablets (2 mg and 4 mg)**

Reversible and selective inhibitor of JAK1 and JAK2 kinases.

Dosage: 4 mg per day until a minimum of 5 days of treatment or until respiratory improvement (maximum 7 days of therapy). The route of administration is oral.

If kidney failure:

- CLCr between 30 and 50 mL / min administer 2 mg per day

- CLCr <30 mL / min do not administer medication

If liver failure, the use of baricitinib is not recommended.

**Risk of Infection**

JAK inhibitors (Ruxolitinib, Tofacitinib and Baricitinib) block the functionality of T cells, reducing the production capacity of pro-inflammatory cytokines, associating a higher risk of infection.

Experience with the use of Ruxolitinib showed that patients with hematological malignancies treated with this drug had a significant reduction in NK cell counts, a fact that led to a higher percentage of infections, the most frequent being urinary tract infections, pneumonia, and varicella zoster virus infection. (VVZ), septic shock and reactivation of tuberculosis disease. Additionally, an increased risk of grade 3-4 neutropenia was also associated with the use of Ruxolitinib (PMID 28007736).

The clinical trials that analyzed the presence of opportunistic infections with the use of Tofacitinib showed a higher risk of developing tuberculosis infection (with the use of Tofacitinib at maximum doses of 10mg / 12h), with previous treatment with isoniazid being a protective factor. Other opportunistic infections identified were esophageal candidiasis, disseminated VZV, Cytomegalovirus infection, and P. jiroveccii pneumonia (PMID 26318385).

Baricitinib is the latest JAK inhibitor approved by the EMA and the data regarding infection come from two phase 2 and 3 clinical trials where the risk of infection was compared with placebo, globally being 3%. The most frequently observed infections were varicella zoster virus (VZV) infections with the use of Baricitinib at high doses of 4mg (PMID 27028914) and mostly not severe.

Taking into account the above data, and according to the European guidelines for infection associated with biological treatments (PMID 29454849), a screening for the following infections is recommended prior to starting treatment with Baricitinib:

-Chronic HBV infection: Anti-HBc, HBsAg and anti-HBsAg should be requested. In case of positive HBsAg, antiviral prophylaxis should be administered and in those patients with positive anti-HBc and negative HBsAg, HBV PCR should be requested to rule out hidden HBV infection. In case of being positive the HBV PCR or meeting criteria of antiviral prophylaxis, it is recommended to consult with gastroenterologist.

- Latent tuberculosis infection: QuantiFERON-TB® should be requested from all patients. In the case of a positive QuantiFERON-TB®, an evaluation by Infectious Diseases Consultant will be requested to rule out active infection by M. Tuberculosis (including directed anamnesis and performance of a chest X-ray if not previously performed). If active disease is ruled out, the indication for treatment of latent tuberculosis infection will be assessed together with the Tuberculosis Unit.

The increased risk of other opportunistic infections such as P. Jirovecii (PJ) pneumonia, disseminated herpetic infection, and invasive fungal infection must also be taken into account, especially in the face of additional immunosuppressive factors (concomitant treatment with steroids, lymphopenia, or prolonged neutropenia). In this context, the indication of anti-PJ, anti-fungal and / or antiviral prophylaxis with Acyclovir will be assessed individually with the Infectious Diseases Consultant. Antibacterial prophylaxis will be performed according to the standard protocols of our center.

**Adverse effects:**

Upper respiratory tract infections, herpes zoster, herpes simplex, gastroenteritis, urinary tract infections; thrombocytosis> 600 x 10 9 cells / l; hypercholesterolemia; sickness; ALT increase ≥3 x ULN.

**9.1.2. Interactions**

- Risk of immunosuppression added with: strong immunosuppressants such as azathioprine, tacrolimus or cyclosporine.

Plasma exposure increased with: probenecid (OAT3 inhibitor with strong inhibition potential), as no tests have been performed

specific interactions, caution when administering leflunomide or teriflunomide (weak OAT3 inhibitor).

**9.1.2.1. Dose limiting toxicity**

Toxicity will be classified according to the National Cancer Institute Common Toxicity Criteria (CTCAEv 5.0)

Grade 3 toxicity. Exception haematological toxicity, alopecia, nausea and vomiting. Any toxicity considered serious at the discretion of the investigator.

**9.1.4.1 Unacceptable toxicity**

It will be defined as that toxicity that, in the investigator's discretion, is considered capable of producing a greater harm than the potential benefit of the treatment. It will be assimilated to dose limiting toxicity.

**9.2. Concomitant treatment**

● Antibiotic therapy only if concomitant bacterial infection is suspected. The antibiotic regimen will be individualized based on clinical suspicion, including standard indications from our center for the management of febrile neutropenia.

● Prophylaxis of thromboembolic disease:

o In patients not previously anticoagulated:

▪ In all patients admitted with SARS-CoV-2: Enoxaparin 40mg / 24h or Bemiparin 3500 IU / d or Tinzaparin 4500 IU / 24h via SC

▪ In high-risk patients (BMI> 30kg / m2), a history of previous episodes of venous thrombosis, high-risk prothrombotic tumors, DD> 1000 ug / L, immobilization: Enoxaparin 40mg / 12h or Tinzaparin 90IU / kg / day via sc

▪ In patients with thrombocytopenia, consultation and follow-up for hemostasis will be performed

o In previously anticoagulated patients:

▪ Uncoagulated patients with LMWH: maintain the usual dose during admission

▪ Patients under treatment with vitamin K antagonists: on admission, change to LMWH after consultation and subsequent follow-up for hemostasis

▪ In any other situation, consultation will be carried out and it will be done for hemostasis

● Remdesivir:

o Consider remdesivir in patients with severe pnemonia (SatO2 <94%) with less than 7 days of onset of symptoms and with supplemental oxygen requirements but not using high-flow nasal goggles, non-invasive or invasive mechanical ventilation or ECMO.

o They must present at least two of the following criteria:

▪ Respiratory rate> 24 rpm

▪ SpO2 <94% at ambient air

▪ paO2 / FIO2 <300 mmHg

o Dose: loading dose on the first day of 200mg intravenously followed by a maintenance dose of 100mg / 24h intravenously from day 2 to day 5.

o Contraindications: severe liver disease (ALT> 5 times the upper limit) and kidney failure (GFR <30 ml / min).

**9.3. Other medication**

The use of medications such as Tocilizumab or interferon will not be allowed in the treatment branch. If treatment with Baricitinib is being received, Tocilizumab will not be administered, since the IL-6 signaling pathway is blocked, which would not have a neutral effect, the only thing that increases would be the economic cost and of a resource that can be used in another patient. In both arms of treatment, if there is respiratory deterioration that requires escalation of respiratory support treatment, the use of corticosteroids is allowed according to the protocol or another treatment that is considered useful in the management of these patients.

The use of tocilizumab will be reserved for patients in the treatment branch at the discretion of the investigator and will be used according to the following criteria:

- Interstitial pneumonia with severe respiratory failure (score = 2)

- Consider tocilizumab in patients who are NOT on mechanical ventilation or ECMO and who are still progressing with corticoid treatment or if they are not candidates for corticosteroids”.

- Mild ARDS (PAFI <300 mmHg) with radiological or gasometric deterioration that meets at least one of the following criteria: CRP> 100mg / LDimerD> 1,000μ / L LDH> 400U / L Ferritin> 700ng / ml Interleukin 6≥40ng / L

- Patient who, according to his baseline clinical condition, would be an ICU tributary

The use of Tocilizumab is not recommended in:

- AST / ALT values ​​greater than 10 times the upper limit of normal

- Neutrophils <500 cells / mm3

- Sepsis due to other pathogens other than SARS-CoV-2

- Presence of comorbidity that can lead to a poor prognosis

- Complicated diverticulitis or intestinal perforation

- Ongoing skin infection

The dose will be that recommended by the AEMPs:

● In patients ≥ 75Kg: 600mg dose

● In patients <75kg: 400mg dose

Exceptionally, a second infusion can be assessed 12 hours after the first dose in those patients who experience a worsening of laboratory parameters after a first favorable response.

Intravenous administration:

-Dilute the prescribed dose in 50-100cc SF 0.9%. Administer immediately after diluting. Infusion times: 1 hour

-Stability of diluted Tocilizumab: 24h at 30ºC

Renal impairment: No dose adjustment required

Hepatic impairment: Caution is advised. There are no data on liver failure.

***Corticosteroids**

If its use is considered, it is recommended in patients who have had symptoms for more than 7 days and who meet all the following criteria:

- need for oxygen support, non-invasive or invasive mechanical ventilation

- acute respiratory failure or rapid deterioration of gas exchange

- appearance or worsening of bilateral alveolar-interstitial infiltrates at the radiological level

In case of indication it is recommended:

- Dexamethasone 6mg / d orally or iv for 10 days

or

- Methylprednisolone 32mg / d orally or 30mg iv for 10 days or

- Prednisone 40mg day p.o. for 10 days

**10. DEVELOPMENT OF THE TRIAL AND EVALUATION OF THE TREATMENT RESPONSE**

**10.1. STUDY SCHEME**

| **EVALUATION** | **BASAL, Day 0** | **Day 2** | **Day 7** | **Day 14 o discharge** | **Day30** |
| --- | --- | --- | --- | --- | --- |
| History | X |  |  |  |  |
| Physical examination | X | X | X | X |  |
| IC obtention | X |  |  |  |  |
| Blood count | X | X | X | X |  |
| Coagulation test (PT, PTT, d-dimer, fibrinogen) | X | X | X | X |  |
| Biochemistry ^a^ | X | X | X | X |  |
| Microbiology ^b^ | X |  |  |  |  |
| RT-PCR SARS-Cov-2 | X |  | X | X |  |
| ECG | X |  |  |  |  |
| Chest XR *o thoracic CT | X | X | X | X |  |
| Biological samples ^,d,e,f^ | X | X | X | X |  |
| Lymphocyte subpopulation | X |  |  | X |  |
| Response evaluation | X |  |  | X | X (phone contact) |

a Ferritin, LDH, CRP, procalcitonin, AST, ALT, bilirubin, Na, K, Cl, Ca, Mg, creatinine, urea, urates, troponin T, albumin, creatinkinase, emogas arterial analysis

b Legionella and pneumococcus antigenuria, sputum, respiratory virus smear, COVID19 PCR, infection screening based on clinical suspicion

c immunological markers: type I IFNs, IFNy, IL-1-a, IL-1b, IL-1RA, IL-6, IL-8, IL-10, IL17, IL-18, IL-33, TFNa, MCP1 / CCL2, TGFb, CD163, sCD25

d inflammation markers: S100A, HMGB1, cytokeratin18, GDF-15, NGAL, TRAIL, TREM-1, Hsp70, Hsp60, chitinase 3-like-1, cytochrome C, IDO, NT-proBNP, TWEAK

and cell death markers: FasL, caspase-3, RIPK3, MLKL, MLKL-p, beclin-1, p62, LC3-II, lamp-1, lamp-2, atg5, atg14, PARP

f pulmonary fibrosis markers: KL-6, SP-A, SP-D, MMP7

**10.1.1. Evaluations before treatment:**

Prior to treatment, a complete evaluation will be performed that will include a history and physical examination. In addition, the following tests will have been performed:

● Hematology: Hemogram, differential count and platelets, reticulocytes.

● Biochemistry: Ferritin, LDH, CRP, procalcitonin, AST, ALT, bilirubin, Na, K, Cl, Ca, Mg, creatinine, urea, urates, troponin T, albumin, creatinkinase, arterial blood gas

● Microbiology: Legionella and pneumococcal antigenurias, sputum culture, respiratory virus smears (RSV, influenza A and B), COVID-19 PCR

● Screening for opportunistic infection according to clinical suspicion. A previously detailed serological study of all patients (HIV, HBV, HCV, VZV, HSV-1 and 2, Quantiferon-TB)

● ECG.

● Chest X-ray or chest CT according to clinical need

● Biological samples to study biomarkers

● Quantification of lymphocyte subpopulations in peripheral blood

**10.1.2. Assessments during the study (day 2, day 7, day 14 or discharge, day 30):**

Throughout the treatment, the following evaluations will be performed:

● Hematology: Hemogram, differential count and platelets, reticulocytes.

● Biochemistry: Ferritin, LDH, CRP, procalcitonin, AST, ALT, bilirubin, Na, K, Cl, Ca, Mg, creatinine, urea, urates, troponin T, albumin, creatin-kinase, arterial analysis gas

● Chest X-ray or chest CT according to clinical need

● Biological samples for the study of biomarkers

● Quantification of lymphocyte subpopulations in peripheral blood

● At day +30 there will be a telephone screening to know the clinical status of the patient. No radiological or analytical tests will be necessary.

**10.2. Obtaining biological samples**

**10.2.1. Samples for future studies:**

Basal (pre-dose): 2 9cc tubes of serum and 2 9cc anticoagulation tubes

At 48h, 2 9cc tubes of serum and 2 9cc anticoagulation tubes

At 7 days: 2 9cc tubes of serum and 2 9cc anticoagulation tubes

Centrifugation protocol and storage until analysis.

**10.3. TOXICITY ASSESSMENT**

Monitoring of patients will be carried out at baseline and after treatment at the following time points:

- Daily vital signs until discharge according to clinical need

- Hematology and biochemistry at day 0, +48 hours, + 7 days, + 14 days or at discharge

- Immune profile day 0, +48 hours, + 7 days, + 14 days or at discharge

Any toxicity equal to or greater than grade 3 (according to CTCAE v.5 criteria) will be recorded

**10.4. Pharmacokinetic and pharmacodynamic analyzes**

Does not apply

**10.5. Withdrawals and dropouts**

A patient will drop out of the study for any of the following reasons:

- If it is considered of interest for the patient to suspend the treatment, according to physician’s criteria.

- If requested by the patient.

- If there is non-compliance with the protocol.

- If it presents an unacceptable toxicity or threat to life.

- Progression of the disease until the need for mechanical ventilatory support or concomitant use of some other agent for COVID19 that is not part of the standard treatment.

- Intercurrent illnesses or other reasons that, in the opinion of the researcher, significantly affect the assessment of the clinical state and that require the interruption of treatment.

**11. ADVERSE EVENTS**

11.1. Definitions

ADVERSE EVENT

An adverse event (AE) is any incidence that is harmful to health in a patient or clinical trial subject treated with a drug, even if it is not necessarily related to such treatment.

ADVERSE REACTION (AR)

It is any harmful and unintended reaction to an investigational drug, regardless of the dose administered.

SERIOUS ADVERSE EVENT AND SERIOUS ADVERSE REACTION

Severe AE (SAE) or serious AR (SAR) are those that at any dose:

- Cause death.

- Put your life at risk.

- Require the hospitalization of the patient or the prolongation of an existing hospitalization.

- Cause permanent or significant disability or disability.

- Cause a congenital anomaly or malformation.

- It is considered medically relevant.

UNEXPECTED ADVERSE REACTION

Any AR whose nature, severity or consequences does not correspond to the Reference Security Information (RSI) is considered unexpected.

SERIOUS AND UNEXPECTED ADVERSE REACTION

Serious Unexpected Adverse Reactions (SUSARs) are considered to be those whose nature, severity or consequences do not correspond to the information contained in the technical sheets of the drugs used.

ADVERSE EVENTS OF SPECIAL INTEREST

The researcher will register in the eCDR and inform the promoter or whoever assumes the tasks delegated by the promoter, the AEs that are considered of special interest as soon as possible and no later than 15 days after he becomes aware of them.

**11.2. Reference safety information**

Investigational Drugs (ID) will be considered in this study: baricitinib

The reference safety information of the study drug corresponds to the reference technical sheet of the investigational drugs: Baricitinib Olumiant®

**11.3. Assessment of adverse events for the safety cohort**

The investigator will systematically monitor and collect all AEs from the first administration of the investigational drug to the subject's final follow-up visit.

All AEs will be recorded in the patient's medical history and in the CRD-e, recording the causal relationship with the trial treatment.

The characteristics of the AEs that have occurred must be recorded as follows in the CRDe:

● Description of the event, start and end dates and severity.

● Intensity according to CTCAE criteria, version 5.0.

● Duration: if it has persisted for a certain time (days, hours or minutes) or continued (if it is still present at the end of the clinical trial).

● Measures taken against the adverse event such as:

- None: No action is applied.

- Medication: Any medication initiated to remedy the adverse event.

- Others: When the measures taken are different from the administration of a drug. For example, physiotherapy, a surgical intervention, etc.

● Measures taken against the study treatments:

- None

- Dose reduction

- Increased dose

- Temporary interruption of medication

- Withdrawal of medication

• Causality with the Investigational Drug:

Related AEs: The temporal relationship of AE with the study medication indicates a possible causal relationship and cannot be explained by factors such as the patient's clinical status or other therapeutic interventions.

Unrelated AE: The temporal relationship of AE with the study medication indicates an unlikely causal relationship, or other factors (medication or concomitant conditions), other therapeutic interventions provide a satisfactory explanation for AE.

• Outcome:

- Recovered

- In recovery

- Not recovered

- Recovered with sequelae

- Deadly

**11.4. Assessment of adverse events for the futility cohort / phase II trial**

The investigator will systematically monitor and collect all AE from the first administration of the investigational drug to the subject's final follow-up visit.

All AAs will be recorded in the patient's clinical history, recording the causal relationship with the trial treatment. However, AEs will only be included in the CRD-e when it is considered:

1) That there is a possible causal relationship with the investigational drug

2) When they are serious.

3) When they are of special interest

Baricitinib

a) Infections

b) Reactivation by herpes zoster virus

The characteristics of the AEs that occurred should be recorded as follows:

● Description of the event, start and end dates and severity

● Intensity according to CTCAE criteria, version 5.0.

● Duration: if it has persisted for a certain time (days, hours or minutes) or continued (if it is still present at the end of the clinical trial).

● Measures taken against the adverse event such as:

- None: No action is applied.

- Medication: Any medication initiated to remedy the adverse event.

- Others: When the measures taken are different from the administration of a drug. For example, physiotherapy, a surgical intervention, etc.

● Measures taken against the study treatment:

- None

- Dose reduction

- Increased dose

- Temporary interruption of medication

- Withdrawal of medication

• Causality with the Investigational Drug:

Related AEs: The temporal relationship of AEs with the study medication indicates a possible causal relationship and cannot be explained by factors such as the patient's clinical status or other therapeutic interventions.

Unrelated AEs: The temporal relationship of AEs with the study medication indicates an unlikely causal relationship, or other factors (medication or concomitant conditions), other therapeutic interventions provide a satisfactory explanation for AEs.

• Outcome:

- Recovered

- In recovery

- Not recovered

- Recovered with sequelae

- Deadly

**11.5. Follow-up of patients with adverse events**

The investigator will systematically monitor and collect AE from the first administration of the investigational drug until the subject's final follow-up visit.

Subjects who present AE, or any abnormal laboratory result that is considered clinically relevant, will be followed up until a satisfactory resolution is reached, until it stabilizes, or until it can be explained by other causes and clinical judgment. indicate that no further evaluations are necessary.

**11.6. Notification of adverse events**

All SAEs (including death), regardless of their relationship with Investigational drugs, must be notified as soon as possible, and never later than 24 hours after knowledge of the presentation of the event, to the person or department responsible for pharmacovigilance

The Investigator will carry out the notification through the SAEs Notification Form, sending it to the e-mail address of the promoter or whoever assumes the tasks delegated by the promoter.

The sponsor or whoever assumes the pharmacovigilance tasks delegated by the sponsor will review the form received and, if applicable, request additional information from the researcher.

The researcher will provide information to the promoter or whoever assumes the tasks delegated by the promoter whenever it is requested and, in any case, when his initial assessment in terms of severity or causality changes. Likewise, all additional information regarding AEs until the end of the study or until its final outcome must be communicated without delay, through follow-up reports following the notification procedure described previously.

Both the first report and follow-up reports will identify patients using the unique patient code and never by name or other identifiable information. The report will also include the study code.

The Investigator is obliged to comply with the legal requirements that are applicable with respect to the communication of SAEs

The promoter or whoever assumes the tasks delegated by the promoter will keep a detailed record of all SAEs and AEs of special interest that are communicated to him by the researchers.

**11.7. Serious and unexpected adverse reactions**

The promoter or whoever assumes the tasks delegated by the promoter will report all suspicions of SUSARs in accordance with current regulations on clinical trials to the AEMPS within a maximum period of 15 calendar days from the moment in which they become known . The competent body of each of the Autonomous Communities where the test is carried out (if applicable) will be notified of the suspicions of SUSARs occurring in the health centers of your Community.

When the SUSAR has caused the death of the patient or endangered his life, the notification will be made within a maximum period of 7 calendar days from the moment in which it is known. Relevant information regarding subsequent events will be completed within eight days.

**11.8. Expedited notification of other relevant safety information**

The promoter or whoever assumes the tasks delegated by the promoter will notify as soon as possible and no later than 15 days after becoming aware of it of any information that could imply a modification of the risk / benefit ratio of the investigational drug or that was of sufficient relevance to recommend modifications in the administration of the investigational drug or in the conduct of the study.

**11.9. Pregnancy**

Subjects will be instructed to report pregnancy if it occurs. In this case the patient will be withdrawn from the trial.

In the event that any pregnancy occurs during the development of the study, the researcher will notify the promoter or whoever assumes the tasks delegated by the notification of SAEs.

The notification will be made using the specific pregnancy notification form, which will be sent by email to the same contact that will receive the notifications from SAEs

**11.10. Report to Researchers**

The sponsor will present the researchers with safety information that could impact the safety of the patients included in the study as soon as possible. The researcher will be informed of the protocol modifications due to security reasons.

**12. ETHICAL ASPECTS**

**12.1. General considerations**

This study will be carried out following the standards dictated by the Declaration of Helsinki (Appendix C), the Standards of Good Clinical Practice and complying with current legislation.

The study will be carried out in compliance with the requirements of the protocol.

Medical assistance to the patient will not be affected by the acceptance or refusal of the patient to participate in the study. No financial or material incentive will be given to the patient. The patient may leave the trial at any time.

If, during the treatment, it is considered that the requirements of the protocol could be detrimental to the patient's health, the patient should be withdrawn from the study.

The investigator and his team are responsible for the medical care of the patients during the study.

**12.1.1 Approval of the protocol by the Ethics Committee for Drug Research.**

Before the start of the study, the protocol, together with the informed consent and the patient information sheet and any relevant amendments, will receive the approval / favorable opinion of the Ethical Committee for Research with Medicines and the Spanish Medicines Agency. Non-significant changes to the protocol will be notified to the Committee and the Spanish Medicines Agency, following current regulations.

**12.2. Informed consent**

The rights, safety and well-being of the trial subjects are the most important considerations and should prevail over the interests of science and society.

The study personnel involved in conducting this trial will be sufficiently qualified by their education, training, and experience to carry out the assigned tasks.

This trial will not use the services of personnel who have been sanctioned / suspended for scientific fraud or clinical malpractice.

Procedures will be implemented to ensure quality in each aspect of the study.

The preparation of the informed consent form is the responsibility of the Investigator. This must include all the elements required by the ICH, the GCPs, and current regulatory directives, and must comply with the Standards of Good Clinical Practice and the ethical principles that have their origin in the Declaration of Helsinki. The consent form should also include a statement that the health authorities will have direct access to the subjects' records.

Before the start of the study, the approval / favorable opinion of the Ethical Committee must be obtained in writing regarding the informed consent form and any other information that will be provided to the subjects.

The patient will express their consent preferably in writing or, if they cannot read, orally before impartial and independent witnesses of the research team, present during the discussion of the informed consent, who will declare it in writing under their responsibility After the patient has granted consent orally, witnesses must personally sign and date the informed consent form indicating that the information provided is accurate, and that the subject understands and has freely given their consent.

The Investigator must provide the patient with a copy of the consent form and the patient information sheet, in non-technical and easily understandable language. The Investigator should allow the subject time to inquire about the details of the study. Then, both the subject and the person who conducted the informed consent discussion will personally sign and date the informed consent. The subject should receive a copy of the informed consent form and any other written information provided to the study subjects prior to their participation in the study.

The informed consent and any other information provided to the subjects must be reviewed whenever new relevant information is available regarding the subject's voluntary participation, and must receive the approval / favorable opinion of the Ethical Committee before its implementation. The Investigator, or the person designated by him, must fully inform the subject of all relevant aspects of the study, and of any new information regarding the subject's willingness to continue their participation in the study. This communication must be documented.

During a subject's participation in the trial, any updates to the consent form or patient information sheet should be provided to the subject.

The subject participating in a clinical trial may or may revoke their consent at any time, without expression of cause and without any liability or damage being derived from it.

**12.3. Confidentiality**

The investigator will ensure that all persons involved respect the confidentiality of any information about the trial subjects. All the parties involved in a clinical trial will keep the strictest confidentiality so that the personal or family privacy of the subjects participating in it is not violated. Likewise, appropriate measures should be taken to prevent unauthorized access to the test data.

The treatment of personal data of the subjects participating in the trial, especially with regard to consent, will comply with the provisions of current legislation (General Data Protection Regulation (EU) 2016/679 and Law Organic 3/2018, of December 5, Protection of Personal Data and guarantee of digital rights).

The data obtained in this study will be reviewed by a monitor or an authorized representative of the health authorities and will be used exclusively to obtain scientific conclusions. The identity of the patients is confidential and will only be recognized by the researcher and his collaborators, the auditors and monitors and inspectors of the competent authorities.

**12.4. Insurance policy**

The promoter of the study has a civil liability policy following current regulations. The policy will be presented prior to the trial.

**13. PRACTICAL CONSIDERATIONS**

**13.1. Investigator responsibilities**

* Ensure that you have enough time to complete the study, with adequate equipment and appropriate facilities until the end of the study, and ensure that you are not conducting other studies that could divert patients away from it.

* Properly complete and keep up to date the data collection notebooks and other study documents.

Investigator Reports: The investigator will submit, at appropriate intervals to the Clinical Research Ethics Committee, accurate and adequate reports on the progress of the study. In the same way, they will present them within the three months following the completion or interruption of the study.

**13.2. Responsibility for the product**

The research team will be responsible for all treatment administration and evaluation throughout the study period.

Product inventory report

The investigator, or the responsible party designated by the investigator, must ensure that the compound is not misused by maintaining dispensing inventories of the study medication.

**13.3. File maintenance**

The researcher will keep the product supply records, data collection sheets and documentation (Note: Documentation is defined as the patient's history, hospital charts, office notes, the patient's diary and all original documents of which information has been obtained to complete the data collection sheets).

These documents must be kept for the maximum period admitted by the hospital, institution or private entity or the period that is longer of the three. The researcher, for a minimum period of 25 years after the interruption or termination of the study, must keep a copy of the subject's identification code, identifying which initials and number of the patient correspond to their real name.

**13.4. Data collection notebooks**

A data collection notebook (CRD) will be designed according to the protocol. Subjects will be identified by a numerical code.

All the required information must be recorded in the data collection notebook. In the event that a data is not available, or it is not applicable, this circumstance must be indicated. Corrections and annotations will be traced in the eCRD

**13.5. Protocol adherence**

Except in emergency situations where appropriate care for the protection, safety and well-being of the study subject requires an alternative treatment, the study will be carried out as described in the approved protocol. Any deviation from the protocol must be documented and justified by the principal investigator of the center.

**13.6. Amendments to the protocol**

The format of the amendments will consist of: (1) front page; (2) reasons for the amendment; (3) list of the parts of the protocols to change, as well as the specific changes. The written amendment must first be approved by the researcher and submitted to the Ethics Committee for drug research. The documentation related to its approval by the Committee (signed by the president or his delegate) must be kept. The promoter will formally present the amendments to the relevant health authorities. It should be noted that when an amendment to a protocol entails a substantial change in the design of the study, its approval must be obtained both by the Ethics Committee and by the Ministry of Health.

**13.7. Study monitoring**

The clinical monitor is obliged to carry out a rigorous follow-up of the study. The clinical monitor will review the data collected in the data collection notebook and will contrast them with the original data from the medical records and other source documents.

**13.8. Valuation variables**

**13.8.1. Main variable:**

**13.8.1.1. Part I: Safety cohort (run-in cohort)**

Rate of serious adverse events due to the combination of baricitinib and treatment at the discretion of the investigator, according to CTCAE version 5.0.

**13.8.1.2. Part II: randomized trial**

Response rate: percentage of cancer patients with pneumonia due to COVID19 who have not progressed to severe respiratory failure with a pO2: FIO2 ratio <100 and who require mechanical oxygen support from the start of therapy until day +14 or the hospital discharge (whichever occurs first).

• It is considered an event if the patient is discharged if the minimum EOSC has been 4.

• It is considered a failure if the minimum ESOC has been 1, 2 or 3.

**13.8.2. Secondary objectives:**

1. Compare mortality within the first 30 days from the start of experimental treatment, in the two treatment arms.

2. Compare the toxicity profile of the two treatment arms.

3. Compare the variability of the immune response due to the experimental treatment vs the standard treatment

4. Evaluate the immunological profile during the study in both arms of treatment.

**13.9. Secondary variables**

- Percentage of cancer patients with pneumonia due to COVID19 who have not required mechanical oxygen support from the start of therapy to day +14 or discharge from hospital (whichever occurs first) in the experimental treatment arm with Baricitinib compared to the treatment branch of the investigator's choice.

- Mortality at +30 days: The mortality rate in each branch will be calculated through the telephone visit on day 30.

- Frequency of toxicities evaluated according to CTCAE V5.0 criteria

- The immune response during the study will be studied using the variables mentioned above.

**14. STATISTICAL METHODS**

There is phase I for this regimen but, since there is no history in these test patients of this regimen, the drug will be tested in a small cohort of filming, based on the principle of achieving a toxicity rate of no more than 33%. The observation period of the dose limiting toxicities (DLTs) will be 14 days. The description of the algorithm is as follows

Step R1. We will start with 6 patients.

− If <1/6 participants have DLTs then the 4 mg daily dose will be determined to be safe.

− If 2/6 participants have DLTs then 6 more patients will be recruited at the same dose:

o If we have 2/12 patients with DLTs then it will be determined that the dose of 4 mg / day is safe.

o If we have> 3/12 patients, the dose will be lowered to 2 mg / day and it will be evaluated (step R2)

− If> = 3/6 participants have DLTs then the dose is de-scaled to 2 mg and it will be evaluated (step R2)

Step R2. Additionally, 6 more patients could be recruited at the dose of 2 mg / day.

− If <= 1/6 of the patients had DLTs with 2 mg / day, then we will determine 2 mg / day as safe.

− If 2/6 participants had DLTs with 2 mg / day, we would additionally add 6 more patients:

o If we have 2/12 patients with DLTs at 2 mg / day then we will determine 2 mg / day as the safe dose

o If we have> = 3/12 patients, we will declare 2 mg / day as unsafe and 2 mg / day will be determined as unsafe, we will consider the treatment as unsafe.

**14.1. Phase II Randomized Trial: Sample Size and Main Analysis**

In this second part, the proportions of patients presenting efficacy of the treatment in the two arms will be compared: treatment with baricitinib vs treatment of the investigator's choice. In this phase an interim analysis will be carried out in which the efficacy will be prematurely compared, and also at the same time a futility analysis based on the predictive power method will be carried out (Spiegelhalter 1986). If, from the analysis of this intermediate step, it is determined that we cannot prematurely demonstrate the efficacy of the experimental treatment arm or its futility, the rest of the patients will be recruited to finally perform the test of comparison of proportions of the main analysis. An overall alpha error of 0.10 will be considered acceptable in the context of a phase II trial.

The main hypothesis test that is proposed is as follows: Let Pe and Pc be the proportion of patients with a scale equal to or greater than 4 in the experimental and control arms respectively. We want to perform the following hypothesis test:

H0: Pe-Pc <0.2 vs H1: Pe-Pc> = 0.2

Taking into account that under the alternative hypothesis Pe = 0.8 and Pc = 0.6, wanting to test a difference of 0.2, it is a question of carrying out a comparison superiority test of two proportions with a global alpha error of 0.10 and a beta error of 0.2. This requires 128 patients, 64 in each arm. Taking into account a 5% loss, 136 patients would be required, 68 per arm. See the main analysis section for more information on the intermediate analysis.

**14.2 Statistical analysis**

**14.2.1. Sample description**

For all the stages of the trial, a description of the sample will be made initially through frequencies of demographic factors, comorbidity (HT, Diabetes, immune diseases, smoking), diagnoses (type of cancer, stage), type of antineoplastic treatment (immunosuppressant, immunotherapy, targeted therapies, with differentiated frequencies for each assigned treatment arm.

Later the consort diagram will be elaborated.

**14.2.2. Definition of analysis populations**

Three analysis populations are defined:

• Safety: All patients who have received at least one dose of baricitinib are included.

• By protocol: All patients who have been able to receive at least the corresponding doses of baricitinib for 7 days will be included.

• By intention to treat: All randomized patients will be included.

Analysis in the safety cohort:

The first result will be the description of the taxi algorithm. Tables of frequency of adverse effects will be made according to CTCAE v5.0 depending on the degree, the relationship with the regimen and the dose modifications they cause. Contingency tables of serious adverse effects will be made according to the demographic variables, the baseline variables, the patient's onco-hematological pathology and their comorbidities. 13.2.3. Phase II efficacy cohort

**14.2.3. Phase II main analysis**

For both the intermediate and the second analysis, the contrast statistic will be based on the student's t distribution with 63 and 127 degrees of freedom. Both analyzes will be performed on the intention-to-treat population. In case of obtaining a p-value lower than 0.05 in the first interim analysis, having recruited 68 evaluable patients, the experimental treatment would be declared promising. Otherwise, the recruitment would continue until the 136 evaluable patients were obtained and if the p-value were below 0.05 we would declare the therapy as promising. If it were above, we will declare the drug as inactive.

To perform the futility analysis we will use the predictive power method described by Spigelhalter et al (1986). This method calculates the posterior predictive probability that the test statistic of the second part of the sample falls in a region that is equivalent to the region of acceptance of the alternative hypothesis of the global statistical test of effectiveness. It will be calculated in the intermediate analysis that it coincides with just 50% of the total sample size, so that f = 0.5. If the power is below 20%, it will be considered, together with the qualitative toxicity analysis, to interrupt the test.

**14.2.4 Secondary analyzes**

To evaluate the factors that influence the efficacy of the treatment, a multivariate logistic regression will be carried out in which the response variable will be having had an EOSC <= 2 vs EOSC> 2 scale as the response variable and which in any case will include the branch variable of treatment as an explanatory variable. This analysis will be performed with the intention-to-treat population. The same analysis will also be carried out with the dichotomous variable requiring mechanical ventilation or not.

To evaluate the mortality rate at 30 days, a multivariate logistic regression will be carried out (death yes vs no as response variable) that in any case will include the treatment branch variable as an explanatory variable. This analysis will be performed with the intention-to-treat population.

To evaluate toxicities, a table of frequencies of occurrences will be made by treatment branch and by relationship reported by the principal investigator. Possible toxic interactions with other treatments will also be investigated using frequency tables. This analysis will be carried out on the security population.

To evaluate both the immune response and the viral load, a repeated measures analysis will be performed taking into account the population per protocol, and will focus on the differences between treatment arms.
